# Supplementary material for: ARL6IP5 Ameliorates α-Synuclein Burden by Inducing Autophagy via Preventing Ubiquitination and Degradation of ATG12
Source: Int J Mol Sci. 2023 Jun 22;24(13):10499. doi: 10.3390/ijms241310499 (PMC10341401; doi:10.3390/ijms241310499)
Supplement: Supplementary file 1 [file ijms-24-10499-s001.zip › ijms-2423483-supplementary.pdf]

Supplementary figure

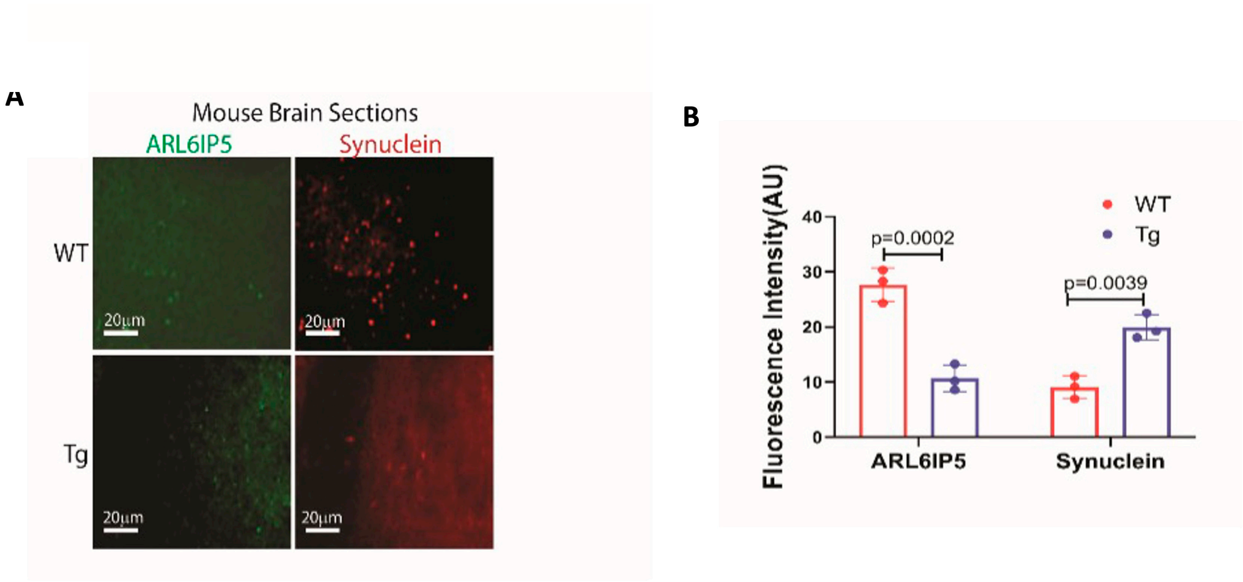

Figure S1. ARL6IP5 was downregulated in Tg-Parkinson's mice (**A**) Representative immunohistochemistry image showing the fluorescence intensity of ARL6IP5 (green) and synuclein (red) in wild type and Tg mouse brain section. (**B**) Graph showing the quantitative analysis of the fluorescence intensity measures in the immunohistochemistry. Results from three independent experiments are presented. *p*-value was calculated using one-way ANOVA with post hoc Tukey test.

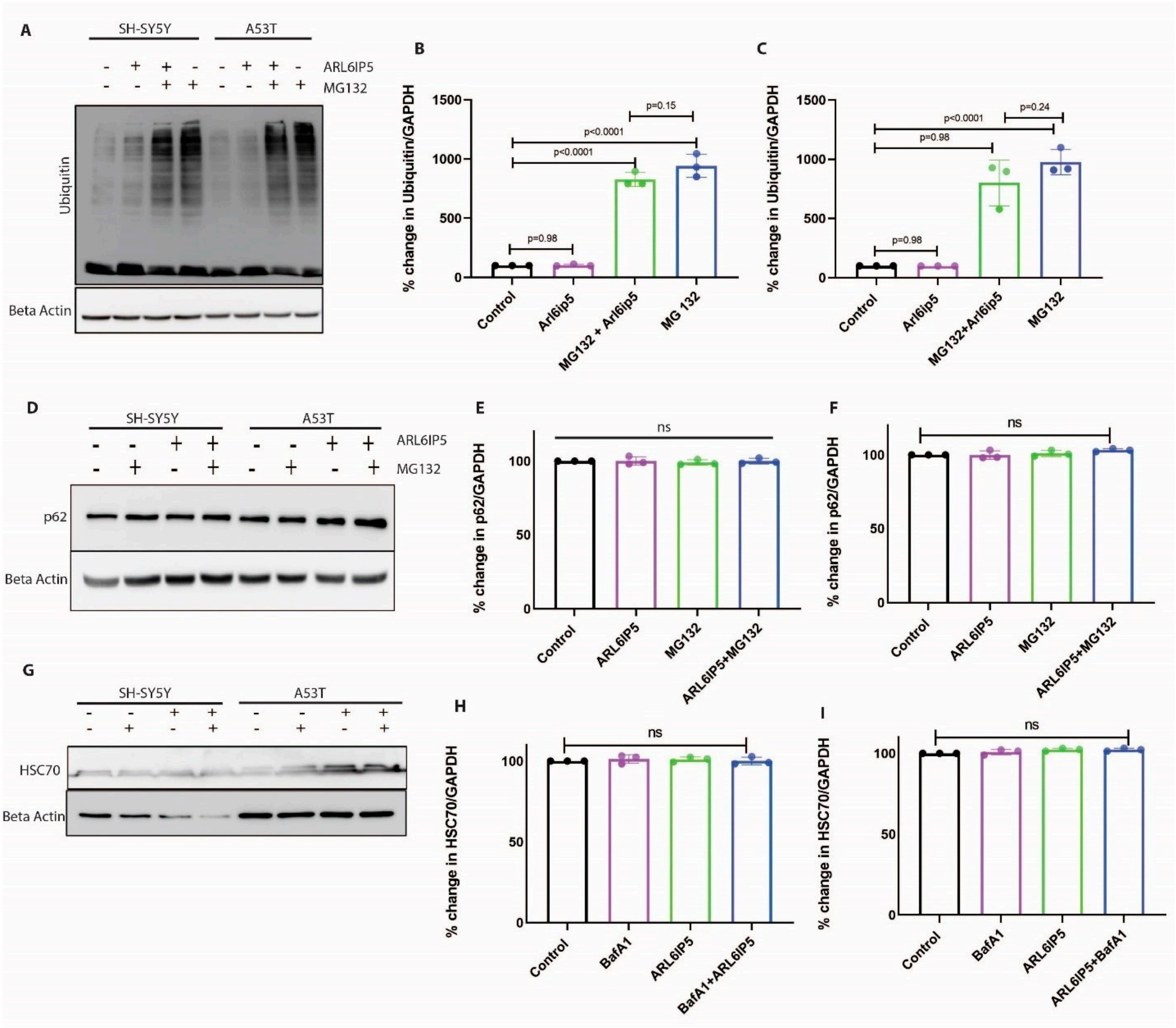

Figure S2. ARL6IP5 does not alter ubiquitination, p62, and HSC70: (**A**) Representative blot showing the level of ubiquitination in SH-SY5Y cells and cells expressing A53T -synuclein (**B**) Densitometric analysis of change in ubiquitination in SH-SY5Y cells. (**C**) Densitometric analysis of change in ubiquitination in SH-SY5Y cells expressing A53T α-synuclein. (**D**) Representative blot showing the level of p62 in SH-SY5Y cells and SH-SY5Y cells expressing A53T α-synuclein. (**E**) Densitometric analysis of change in p62 in SH-SY5Y cells. (**F**) Densitometric analysis of change in p62 in SH-SY5Y cells expressing A53T α-synuclein. (**G**) Representative blot showing the level of HSC70 in SH-SY5Y cells and expressing A53T α-synuclein (**H**) Densitometric analysis of change in HSC70 in SH-SY5Y cells. (**I**) Densitometric analysis of HSC70 in SH-SY5Y cells expressing A53T α-synuclein. Results from three independent experiments are presented. *p*-value was calculated using one-way ANOVA with post hoc Tukey test.
